# What’s a Weed? Knowledge, Attitude and Behaviour of Park Visitors about Weeds

**DOI:** 10.1371/journal.pone.0135026

**Published:** 2015-08-07

**Authors:** Michael Ansong, Catherine Pickering

**Affiliations:** Environmental Futures Research Institute, Griffith University, Gold Coast, Queensland, Australia; University of New South Wales, AUSTRALIA

## Abstract

Weeds are a major threat to biodiversity globally degrading natural areas of high conservation value. But what are our attitudes about weeds and their management including weeds in national parks? Do we know what a weed is? Do we consider weeds a problem? Do we support their management? Are we unintentionally spreading weeds in parks? To answer these questions, we surveyed visitors entering a large popular national park near the city of Brisbane, Australia. Park visitors were knowledgeable about weeds; with >75% correctly defining weeds as ‘plants that grow where they are not wanted’. About 10% of the visitors, however, provided their own sophisticated definitions. This capacity to define weeds did not vary with people’s age, sex or level of education. We constructed a scale measuring visitors’ overall concern about weeds in parks using the responses to ten Likert scale statements. Over 85% of visitors were concerned about weeds with older visitors, hikers, and those who could correctly define weeds more concerned than their counterparts. The majority think visitors unintentionally introduce seeds into parks, with many (63%) having found seeds on their own clothing. However, over a third disposed of these seeds in ways that could facilitate weed spread. Therefore, although most visitors were knowledgeable and concerned about weeds, and support their control, there is a clear need for more effective communication regarding the risk of visitors unintentionally dispersing weed seeds in parks.

## Introduction

Weeds in national parks are a major threat to biodiversity conservation. They compete with native plants for resources such as moisture, light and nutrients contributing to reductions in the populations of native species and, in some cases, increasing the risk of species extinctions [1–4]. Weeds also have major economic impacts; including reducing the quality of ecosystem services provided by national parks, while increasing management costs [1,4–6]. Controlling weeds in parks and other natural areas is expensive, in Australia for example, the cost of controlling environmental weeds was estimated at over $AU20 million (2001–2002 financial year) [6].

The establishment of protected areas, such as national parks, is not only the major mechanism for the conservation of biodiversity but many are also important destinations for tourism and recreation [7–10]. Visiting parks contributes to people’s quality of life including by providing a diversity of physical, psychological and social benefits [7,10]. Unfortunately, there are negative environmental impacts from park visitation, including an increased diversity and abundance of weeds [11–13]. Partly, this is because weeds benefit from disturbance, including that associated with the construction, maintenance and use of different types of visitor infrastructure [14–17]. As a result, weeds dominate the edges of roads, tracks and car parks in many national parks [3,15,16,18,19]. Visitors can also unintentionally introduce and spread new weeds, with a wide diversity of weed seeds found on vehicles and clothing [16,19–22]. For instance, seeds from 626 species of plants have been collected from cars, nearly all of which (96%) are considered weeds somewhere [23]. Seeds from 449 species have been found on clothing, of which 87% are considered weeds somewhere [24]. Animals used for recreation in parks can also spread weed seeds, with seeds from 249 species (99% weeds) able to germinate from horse dung [25].

As a result public support for park management in general and for weed management in particular, is important [26–28]. Visitors often financially support weed management in parks directly via the payment of park fees, and/or indirectly via taxes that are subsequently used for park management [29,30]. Support could potentially include changes in behaviour so visitors are unlikely to carry and disperse weed seeds. This includes ensuring that they do not carry seeds on clothing or vehicles when they arrive at a park as well as staying on trails. As support for these types of measures depends on knowledge [26–28,31], we need to understand if park visitors know what weeds are, if they are concerned about weeds in parks, and if they are likely to have seeds on their clothing when entering parks. These questions are difficult to answer as very few studies have assessed the knowledge of, and attitudes towards weeds among park visitors.

The few more general studies of community attitudes to weeds and invasive plant species in general have found some interesting patterns. A study that assessed community knowledge of weeds by surveying residents of Montana in the United States of America, found that most people considered weeds a serious or very serious problem and could name a problem associated with weed invasions [27]. They also knew of different ways that weeds could spread, however, many could not identify ways to prevent the spread of weeds. More recent community studies in Scotland [31] and the United States of America of local park users, different types of tourists and conservation professionals in Spain [32], and tourists in South Africa [29] found that most people could define invasive species but knowledge varied depending on their background. Conservation professionals and nature tourists, for example, were more knowledgeable about invasive species than general tourists and local park users [32]. Those with higher levels of education were also more likely to be aware of invasive species and their impacts [28]. There was a general willingness among people to support weed control, eradication programs and conservation, with males, older people, and those who had previously heard of control and eradication projects, more willing to support such programs than their counterparts [31].

Reflecting the need to better understand the social dimensions of weed management in parks, we assessed visitor’s knowledge, attitudes and behaviour regarding weeds in a large, high conservation value and popular national park. Specifically we assessed: 1) socio-demographic data about visitors and their use of the park; 2) their knowledge of weeds; 3) their attitudes about weeds and weed management in general; and 4) their attitudes and behaviour specifically in relation to seeds on clothing. We assessed if survey responses varied depending on socio-demographic characteristics. Understanding visitor’s knowledge, attitudes and behaviour, is important for the development of effective environmental educational programs including weed management in national parks.

## Methods

### Data collection

**Fig 1:**
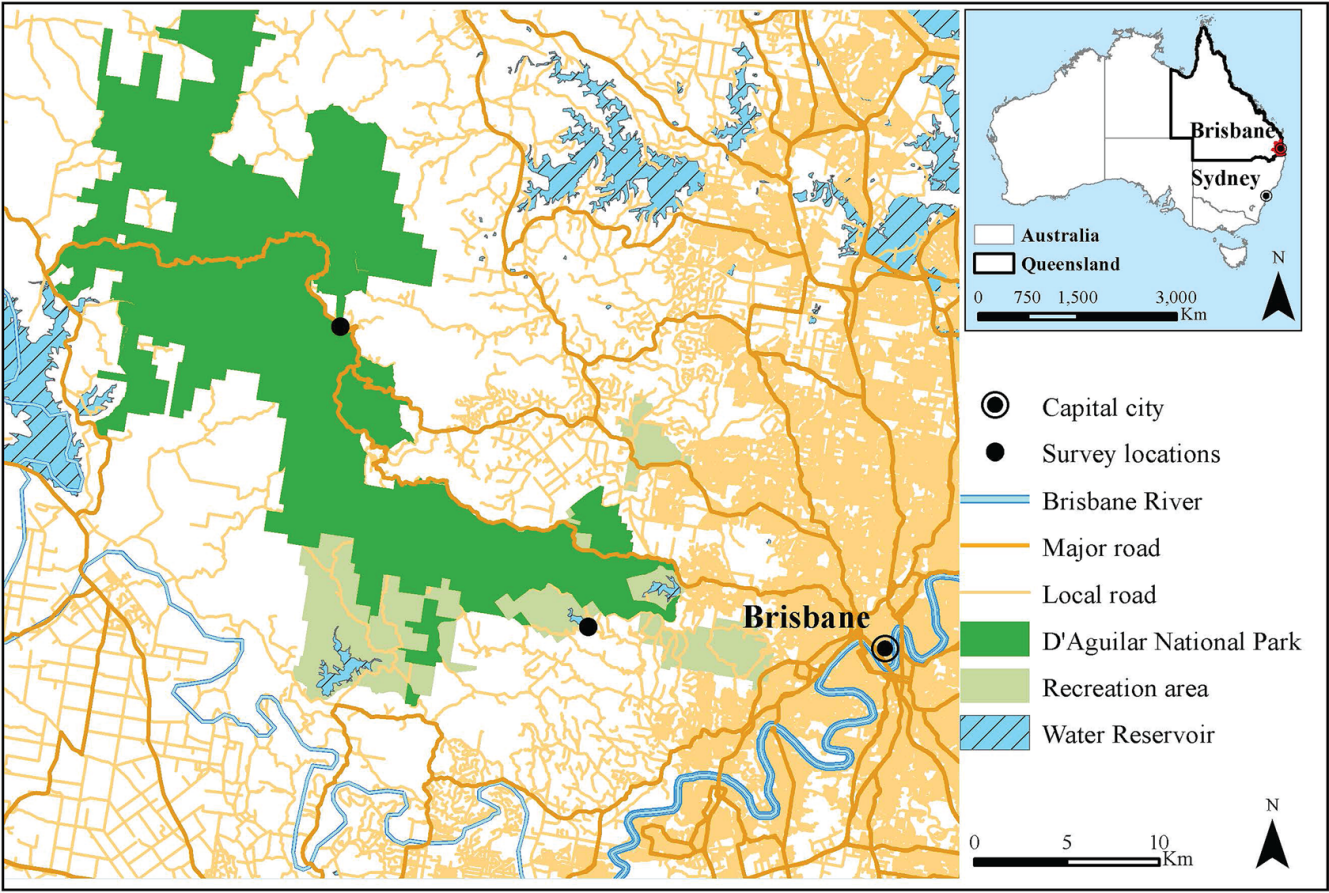
Survey locations in D’Aguilar National Park, close to Brisbane, in south-eastern Australia.

Visitor’s knowledge, attitudes and behaviour regarding weeds were assessed using a structured survey in D’Aguilar National Park (36,000 ha), the largest national park close to the city of Brisbane (population two million) in south-eastern Australia (Fig 1). D’Aguilar National Park was established principally for conservation (e.g. IUCN Category II Park), and it is also used for a wide range of recreational activities, with bushwalking, running and mountain biking the most popular [33,34]. At least thirty species of environmental weds occur in the Park, mostly along the edges of recreation trails [16]. Griffith University Human Research Ethics Committee approved this study (Permit: ENV/50/13/HREC) and the Queensland Department of National Parks, Recreation, Sport and Racing granted the permission to conduct the study in the park.

Visitors to the Park were surveyed at the two main high use entry sites (Gold Creek car park and Maila car park), close to Brisbane (e.g. ~12 km from the city centre) during periods of high usage in the summer school holidays [33,34]. The interviewers approached all visitors on the edge of the car park, close to the start of recreation trails, and after introducing the project and obtaining respondents' consent; participants were provided with a self-completion questionnaire. They were first provided with a printed document which included background material about the survey and a consent form. The document had information about who was conducting the research, a summary of the project description, why the interviewee was asked to participate, the confidentiality and privacy of the data collected, and also volunteer participation in, and possibility of withdrawal from the survey at any time. The contact details of the researchers and Griffith University Ethics Manager were also provided. If they agreed to the surveyed and sign (by ticking a box) the consent form, visitors were then provided with the questionnaire, which they completed themselves.

Visitors over 15 years old who gave consent were included in the survey. Visitors 15 years or younger were not included as they may not be able to adequately understand the survey questions or concepts presented in the questionnaire. Griffith University Human Research Ethics Committee approved the consent procedure and the age group included in the study. During the four days of the survey 161 visitors used the trails from the car parks. We approached 145 people and asked them if they were willing to be surveyed. Of these, 112 completed the survey resulting in a response rate of 77%.

The questionnaire consisted of close-ended and open-ended questions to collect data on visitor: 1) socio-demographic characteristics and their use of the Park; 2) knowledge of weeds; 3) attitudes about weeds and weed management in general; and 4) attitudes and behaviour in relation to seeds on their clothing. General socio-demographic and visitation data included age, sex, education level, the type of recreational activity people were engaging in, if they were visiting on their own or in a group, income level, means of transport, time taken to reach the Park, number of hours they expected to spend in the Park, where they came from (home or other place) and how often they visited the Park.

Knowledge of weeds was measured by the ability of respondents to correctly select the statement that they felt best described weeds from five definitions provided, or provide their own definition. The five definitions provided were: 1) ‘plants that grow where they are not wanted’; 2) ‘plants that are used for medicinal purposes’; 3) ‘plants that grow in gardens and agriculture lands’; 4) ‘plants that grow around road sides and water ways’; and 5) ‘I am not sure’.

We are using the term attitudes here to mean ‘psychological tendencies expressed by evaluating a particular entity with some degree of favour or disfavour’ [35]. Attitudes were measured using a psychometric scale (Likert scale) that provided a range of responses to each statement from which people could choose the option that best coincided with their view or belief [36]. The attitudinal statements measured visitor’s thoughts about weeds (cognitive), how they feel about weeds in the Park (affective) and to what extent they were willing to support management of weeds (behavioural intentions).

To assess visitor’s attitudes, we used Likert-scale statements each with five categories of responses; from strongly disagree (1) to strongly agree (5). This included ten statements relating to their level of concern regarding weeds in the Park (Table 1) and one statement about their willingness to fund weed management (“Money should be spent to control/eradicate weeds in the Park”). They were then asked four related questions regarding seeds on their clothing. Specifically they were asked about their: 1) perceptions about the problem (e.g. “Visitors do unintentionally introduce plant seeds in parks” Likert-scale), 2) direct experience (“Have you ever found seeds on your clothing [footwear, socks, skirt, trousers, etc], before, during or after walking through this or another park” Yes/No question), 3) response (“How did you dispose of the seed(s)” Open-ended question), and 4) support for behaviour to reduce the risk in the future (“I will support cleaning of visitor’s clothing before entering parks” Likert-scale).

**Table 1:** Loadings of the dimensions from the Likert-like statements used in formulating the two attitude scales and the percentage of visitors who agreed and disagreed with each statement. The extracted dimensions and their eigenvalues are from the Linear Principal Component Analysis rotated using Oblimin rotation (delta = 0) with Kaiser Normalization. Items with only loadings > 0.5 (bolded and shaded) were included in the composite attitude variable created for subsequent analysis.

			% of respondents		
Statements	Rotated dimension loadings	N	AG	Neutral	DA
Money should be spent to control/eradicate weeds in the Park	**0.775**	112	65	23	12
*The environmental threats of weeds have been exaggerated	**0.769**	112	16	37	47
*Weeds in this Park are of concern to me	**0.759**	112	40	41	19
*We don’t have to worry about weeds because the ecosystem will balance itself	**0.750**	112	13	26	61
*Weeds have as much right to exist in this Park as all other plants	**0.664**	112	21	20	59
*The welfare of people should come before thinking of weed eradication	**0.631**	111	27	26	47
*I will support the introduction of non-native plants into the Park if they benefit visitors	**0.583**	111	17	23	60
*Weeds occur naturally in the Park	0.456	112	51	16	33
*Eradication of weeds will affect the aesthetic beauty of the Park	0.367	110	22	19	59
I will do anything to preserve the plants in this Park	0.350	112	56	33	11
Eigenvalue	4.0				
% of variance explain	40.0				
#Cronbach’s alpha (Seven items)	0.83				

Note: N is the number of visitors who responded to the statement; AG is the sum of the percentages of Agree and Strongly Agree while DA is that of Strongly Disagree and Disagree.

*Negative statement with scores reversed.

# The Cronbach’s alpha is based on the seven items selected (bolded and shaded) for subsequent analysis.

### Data analysis

Data from the surveys (S1 Dataset) were analysed in Excel and the statistical program SPSS using a combination of descriptive and inferential statistics. Socio-demographic data about the visitors and their use of the Park were summarised and tabulated. Associations among the socio-demographic data and park use patterns were tested using cross tabulation with chi-square tests of independence. We calculated the percentage of visitors who correctly defined what a weed was, and performed chi-square tests of independence to determine which socio-demographic variables were associated with visitor knowledge of weeds. Visitor responses about their willingness to fund weed management, their perceptions about the problem, and support for behaviour to reduce the risk in the future, were converted into categorical variables (Agree, Neutral and Disagree) and chi-square tests used to assess associations among them and the socio-demographic variables. The associations between direct visitor responses and socio-demographic variables were also tested with cross tabulation.

Principal Component Analysis (PCA) was used to construct an attitudinal scale that can be used to assess visitor’s overall level of concern about weeds in the Park. The oblique rotation, direct oblimin (delta = 0), with Kaiser Normalization [37] was used as the statements are expected to be related. We specified only one component, which accounted for about 40% of the explained variance (Table 1). The data were suitable for PCA analysis as evident by KMO measure of sampling adequacy of 0.796 with non-identity population correlation matrix (Bartlett’s Test of Sphericity = 347, df = 45, p < 0.001). The internal consistency reliability coefficient (α) or Cronbach’s alpha, which explains whether a group of variables measures a single latent phenomenon or one underlying construct [37] was significantly improved when we retained only statements with loadings above 0.5. The result was similar when we tested other robust analysis such as the Categorical Principal Component Analysis.

An attitude scale (concern scale) was constructed that used the seven statements with loadings above 0.5 (Table 1). The attitude scale (concern scale) shows the overall distribution of each individual’s scores on the same metric as the original individual statements. The concern scale was calculated by summing the raw scores (1–5) of the seven individual statements to form a composite test score (Table 1). All negatively worded statements were reversed before the summation. The scale ranged from 1–35, with a score >17.5 indicating that the visitor was concerned about weeds in the park and vice versa for scores <17.5. Using the scale as dependent variable, we tested the effect of different socio-demographic factors, knowledge and behaviour of visitors, on their concerns about weeds using One-way ANOVAs.

## Results

### Socio-demographics of visitors

Most visitors were highly educated (80% university/tertiary education) and there were more men (65%) than women visiting the Park (Table 2).

**Table 2:** Socio-demographic characteristics of the 112 park visitors surveyed in D’Aguilar National Park, south-eastern Queensland, Australia; n = number of respondents.

Variable	n
Frequency of visit	
Regular	17
Occasional	62
First visit	32
Education level	
Tertiary	86
Others	22
Sex	
Female	35
Male	67
Age	
< 25	19
25–44	46
> 44	45
Main activity	
Hiking/bush-walking	65
Running/jogging	19
Others	26
Means of transportation	
Vehicle	103
Foot	9
Time spent in park	
≤ 2 hours	64
≥ 2 hours	47
Where visitors came from	
Home	97
Other	13
Time travelled to the Park	
< 30 min	61
≥ 30 min	51
Income per month (AU$)	
< 8000	77
≥ 8000	23

Visitors were mainly over 25 years old (83%), with those older than 44 years more frequent visitors (20%) than those between 25–44 years (15%). The majority (68%) of visitors had monthly incomes ≤ AU$ 8000 (Table 2).

Most visitors had been to the Park before, with over half (55%) ‘occasional visitors’, visiting a few times per month, or a few times per year. A few, 15%, were more regular visitors coming either several times a week or daily. Over one-quarter (29%), however, were first time visitors to the Park (Table 2). Many (58%) spent less than two hours in the Park (Table 2). Almost all had come from home (88%); most came by car (86%), with only 8% arriving on foot and the rest by other means, mostly on mountain bikes. For those who came by car, half (51%) travelled for less than 30 minutes, 44% travelled for 30–60 minutes, and the rest travelled for over an hour. Many (57%) were going hiking/bushwalking, 17% were going running/jogging while the rest engaged in other recreational activities including bird watching, swimming and cycling (Table 2). The runners/joggers were more regular visitors to the Park than those undertaking other activities (χ^2^ = 20.16, df = 4, p < 0.001). The majority of hikers were older than 44 years, runners/joggers were mostly from 25 to 44 years old, while those younger than 25 were more likely to engage in other activities (χ^2^ = 10.77, df = 4, p = 0.029).

### Knowledge of weeds

Most visitors (75%) were able to effectively define the term ‘weed’ from the options provided, by identifying weeds as “Plants that grow where they are not wanted”. Their ability to define this term was not associated with any of the socio-demographic variables including frequency of visit (χ^2^ = 0.03, df = 2, p = 0.985), age (χ^2^ = 0.53, df = 2, p = 0.769), sex (χ^2^ = 0.86, df = 2, p = 0.355) or level of education (χ^2^ = 0.01, df = 2, p = 0.739). In addition, around 10% of visitors provided their own, often quite sophisticated, definitions of weeds including: “introduced species, be it regional country of origin”, “a plant growing outside its natural environment”, “non-indigenous plant species in context, that may be invasive in a particular ecosystem”, “unwanted plants”, “invasive species”, “plants that grow aggressively and are hard to control”, “a plant which causes a detrimental effect to the local ecosystem”, “plants not native to their environment”, “plants growing in areas where they do not occur naturally but are well suited to conditions and multiply rapidly”, “a species that has grown at the expense of other flora and fauna” and “plants which cause negative environmental or economic impacts that are not native to a location”.

### Visitor’s level of concern about weeds in the Park

Overall, visitors were concerned about weeds in the Park, with the mean attitudinal score of 24.9 ± 0.5 on a scale from zero (not concerned) to thirty five (very concerned). There were 92% of people with a score above the neutral value of 17.5 and only nine people with values less than 17.5. While most visitors agreed with individual attitudinal statements suggesting that the Park needs to be protected from weeds, there were a few who agreed with statements suggesting that weeds pose no threat to the Park because they are part of the natural ecosystem. For example, while 65% agreed that ‘money should be spent to control/eradicate weeds in the Park’ 13% agreed that ‘we don’t have to worry about weeds because the ecosystem will balance itself’ (Table 1). There were also many visitors who were neutral about some attitude statements. For instance, when asked whether weeds in the Park were of concern to them, 41% selected the neutral option (Table 1).

Older visitors were more concerned about weeds than younger visitors (Table 3). Hikers were also more concerned than runners/joggers. Similarly, visitors who were able to correctly define weeds were more concerned about weeds than their counterparts. There were no other significant associations between socio-demographic and visitation data with attitudes about weeds.

**Table 3:** Results of One-way ANOVAs showing the effects of socio-demographic characteristics of visitors on their attitudes towards weeds measured using a concern scale constructed from responses to seven Likert like statements obtained using Principal Component Analysis. P values in bold are significant at alpha < 0.05.

Variable	n	Mean ± SE	df	F	P
Frequency of visit			2	1.741	0.180
Regular	17	24.1 ± 1.2			
Occasional	62	25.8 ± 0.7			
First visit	33	23.6 ± 0.9			
Education			1	1.686	0.197
Tertiary	87	25.2 ± 0.6			
Others	22	23.5 ± 1.1			
Sex			1	1.043	0.310
Female	36	24.2 ± 0.9			
Male	67	25.4 ± 0.7			
Age			2	3.115	**0.048**
< 25	19	23.3 ± 1.1			
25–44	47	24.1 ± 0.8			
> 44	45	26.4 ± 0.8			
Main activity			2	3.850	**0.024**
Hiking/bush-walking	65	25.7 ± 0.7			
Running/jogging	19	21.9 ± 1.0			
Others	27	25.5 ± 0.9			
Define weeds			1	5.877	**0.017**
Yes	82	25.4 ± 0.6			
No	23	22.4 ± 1.1			

### Visitor’s willingness to support spending money on weed management

Over two thirds (65%) of visitors agreed that money should be spent controlling and/or eradicating weeds in the Park. Sex (χ^2^ = 1.36, df = 2, p = 0.587), frequency of visit (χ^2^ = 1.14, df = 4, p = 0.888), activity (χ^2^ = 1.38, df = 4, p = 0.849), age (χ^2^ = 3.59, df = 4, p = 0.465) and level of education (χ^2^ = 3.70, df = 2, p = 0.157) did not influence their answer to this question. The ability to define a weed appeared to influence people’s opinion about whether money should be spent on weed management (χ^2^ = 6.22, df = 2, p = 0.045). Almost 70% of visitors who could correctly identify a weed were in favour of spending money on managing weeds, compared to 46% of those who could not define weeds appropriately. Visitors who supported spending money on managing weeds also tended to be more concerned about weeds in the Park than their counterparts (p < 0.001).

### Visitor’s attitudes and behaviour regarding seeds on clothing

**Fig 2:**
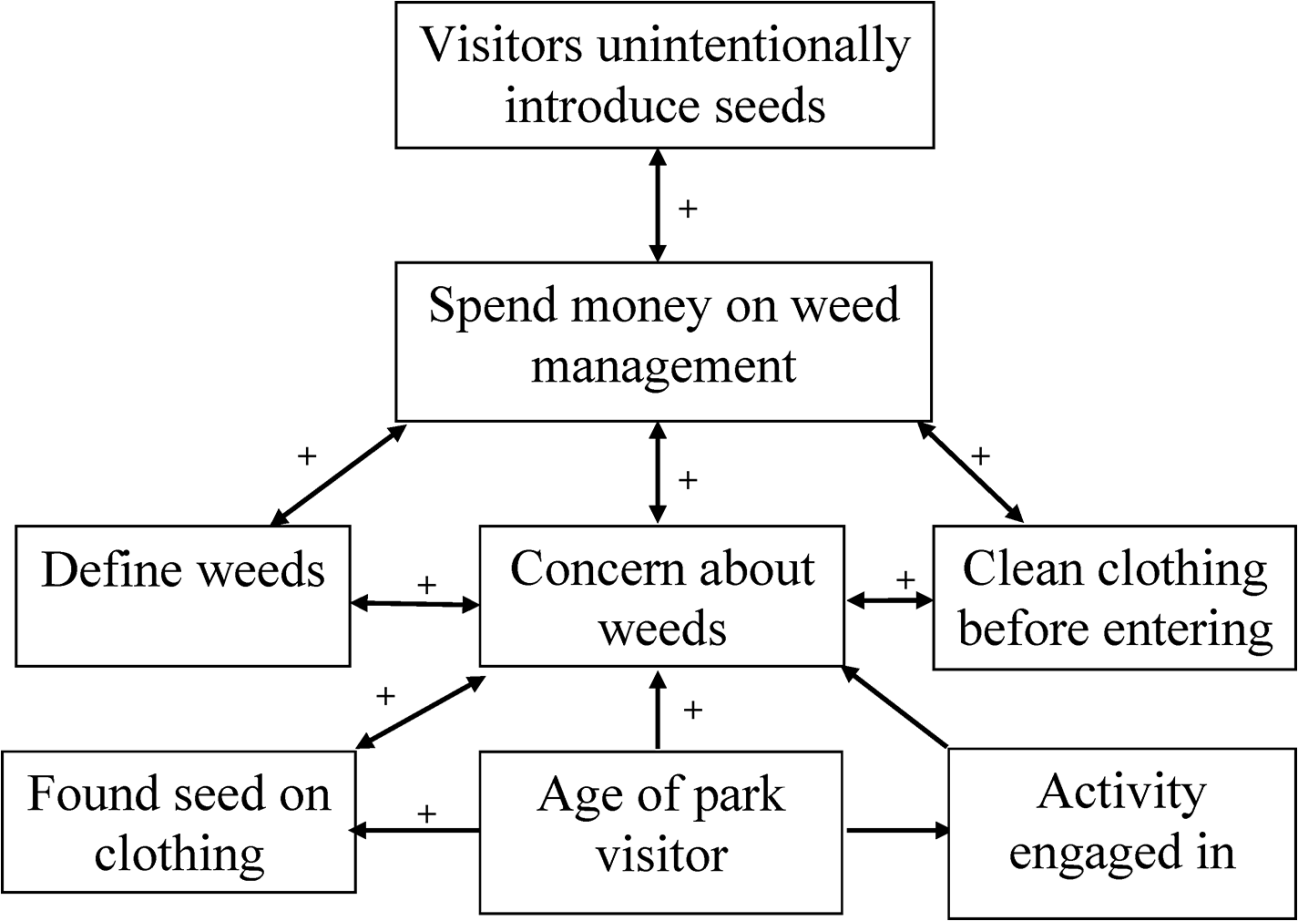
Summary showing significant interactions among all the variables found in the study regarding visitor concerns about weeds and their management in a peri-urban national park in the south-eastern Queensland region. + positive interaction, where if the visitor gave a positive response to one statement, they were more likely to give a positive response to the other statement. For age this means that older visitors were more likely to agree with the statement.

Most (75%) visitors surveyed thought that visitors unintentionally introduced seeds into parks. There was no association between their answer to this question and socio-demographic variables including age (χ^2^ = 2.70, df = 4, p = 0.609), sex (χ^2^ = 1.16, df = 2, p = 0.325), type of activity (χ^2^ = 2.70, df = 4, p = 0.609) level of education (χ^2^ = 2.25, df = 2, p = 0.325) and their level of concern about weeds in the Park (F_(2, 107)_ = 1.58, p = 0.210). The more visitors agreed that people unintentionally dispersed seeds in parks, however, the more likely they were to agree that money should be spent managing weeds in the Park (χ^2^ = 19.58, df = 4, p = 0.001) (Fig 2).

When asked about seeds on their clothing, 63% of visitors reported finding seeds attached to their clothing (footwear, socks, skirt, trousers, etc) before, during, or after visiting the Park. Those able to appropriately define weeds, and those that could not, were equally likely to have found seeds on their clothing (χ^2^ = 0.003, df = 1, p = 0.958). There were also no differences depending on the type of activity they were engaged in (χ^2^ = 0.40, df = 2, p = 0.817), frequency of visits (χ^2^ = 0.50, df = 2, p = 0.778), sex (χ^2^ = 0.64, df = 1, p = 0.424), or level of education (χ^2^ = 0.38, df = 1, p = 0.538). However, older visitors were more likely to have found seeds on their clothing than younger visitors (χ^2^ = 9.07, df = 2, p = 0.011). For example, while 80% of those over 44 years old reported having found seeds on their clothing, only 47% of those younger than 25 years found seeds. Also, compared to the mean response, visitors who reported finding seeds on their clothing were more likely to be concerned about weeds in the Park (F_(1, 109)_ = 4.14, p = 0.044) (Fig 2).

Almost half (49%) of those surveyed support the idea that visitors should clean their clothing before entering the Park, 30% were neutral about this issue while 21% did not agree with the statement. These responses did not vary with socio-demographic variables including age (χ^2^ = 6.34, df = 4, p = 0.175), sex (χ^2^ = 1.87, df = 2, = = 0.393), type of activity (χ^2^ = 4.01, df = 4, p = 0.404), or level of education (χ^2^ = 1.69, df = 2, p = 0.430). Support for cleaning clothing before entering the Park was also unrelated to whether the visitor had found seeds on their clothing (χ^2^ = 3.02, df = 2, p = 0.221). Those who agreed that visitors need to clean their clothing, however, were more likely to agree that money should be spent managing weeds (χ^2^ = 20.11, df = 4, p < 0.001), and were more concerned about weeds in the Park (F_(2, 109)_ = 12.84, p <0.001), than their counterparts.

When asked an open question about what they do with any seeds they find on their clothing, there was a variety of responses. We grouped these into methods that were unlikely to spread seeds in the Park (disposing of the seeds in a bin, removing seeds by washing clothing and/or removing seeds outside the Park), compared with methods that were more likely to result in seeds being left in the Park such as “brush and dust before leaving area”, “brush onto the ground”, “shake shoes before entering car”, “brush them off wherever I am”, “discard carelessly”, or “pick it and leave it at the place I checked”.

Overall, 41% of visitors indicated that they dispose of seeds in the Park. Those who reported finding seeds on their clothing in the Park were more likely to safely dispose of seeds (60%) compared to those who have not found seeds on their clothing (54%). The way in which visitors dispose of seeds was not associated with their age (χ^2^ = 0.1, df = 4, p = 0.816), sex (χ^2^ = 0.91, df = 1, p = 0.341), type of activity (χ^2^ = 1.81, df = 2, p = 0.405), or level of education (χ^2^ = 0.81, df = 2, p = 0.369). It also did not vary with their attitude to spending money on managing weeds in the Park (χ^2^ = 0.29, df = 2, p = 0.867), or support for cleaning visitors’ clothing before entering the Park (χ^2^ = 1.68, df = 2, p = 0.433). The level of concern of those who indiscriminately dispose of seeds was also not significantly different from those who do not (F_(1, 97)_ = 0.03, p = 0.870), with both groups very concerned about weeds in the Park.

## Discussion

Understanding the knowledge, attitudes and behaviour of visitors is important when developing park management strategies and for conservation in general [27,38,39]. We found that knowledge of weeds and concerns about their impacts were high among visitors to a popular national park. Visitors were also willing to support the spending of money on weed management. Although most also supported behaviour such as cleaning clothing before entering D’Aguilar National Park to reduce weed spread in the future, the majority also reported having found seeds on their clothing, with many of them disposing of these seeds in ways that could contibute to the introduction and spread of weeds in the Park.

The considerable ability of visitors to appropriately define weeds is consistent with several other studies that also found that people are often quite knowledgeable about invasive species, especially those who engage in outdoor recreation activities [27,32,38]. In a similar study, for instance, all nature-based tourists surveyed knew the meaning of invasive species [32]. This high level of knowledge about invasive species has been linked to their direct involvement and/or contact with nature [28,32]. Unlike previous studies, however, where knowledge of invasive species was positively associated with education level [28,32], in our study, knowledge of weeds was not significantly related to level of education. This may be due to the predominance of highly educated people surveyed in D’Aguilar National Park Park, with over 80% of the visitors with university level education compared to an average of 19% for the Australian population overall [40].

Visitors’ knowledge about weeds was positively associated with their concern about weeds in the Park with those able to define a weed more concerned about weeds in the Park than their counterparts. Previous studies have also reported that visitor’s knowledge and awareness of weeds and invasive species in general increases their level of concern about invasive species, and support for projects that seek to control/eradicate them [31,32]. In previous studies, when public knowledge and awareness about invasive species were increased through campaigns, there was an increase in concern and support for eradication projects [31,38]. In the current study, visitors who were more knowledge about weeds were also more likely to be willing to financially support the management of weeds in parks.

Older visitors were more concerned about weeds than their younger counterparts. This is similar to the result of Bremner and Park [31] who observed that older people were more willing to support the management of invasive species. Older people often develop greater attachment to a place than their younger counterparts [41]. The older visitors surveyed in D’Aguilar National Park could, therefore, have developed a stronger attachment to the Park, increasing their concern about the weeds in the area. Also, older visitors were more likely to have found seeds on their clothing, which in turn could have added to their concern about weeds (Fig 2).

There were some differences among visitor’s attitudes to weeds based on the type of recreational activity they were engaged in. Although previous studies have found differences in environmental concern among visitor groups, they were usually between people engaged in non-motorised activities and those who prefer motorised activities. Visitors who prefer non-motorised activities were usually more pro-environmentalist than those who prefer motorised activities [42–44]. In our study, all the people surveyed were engaged in non-motorised recreation activities, and nearly all of them were concerned about weeds in parks. However, there were some differences, with hikers more concerned than runners about weeds. Hikers tended to be older than runners, and this may have resulted in their greater concern for the reasons outlined above.

The levels of concern about weeds in the Park were similar for men and women. Differences between men and women in relation to conservation issues depend on the issue assessed [31,45–47]. For example, when the attitude of the public about wildlife conservation in the United States was assessed, women were more concerned than men [46]. A study evaluating attitudes about invasive species in Scotland, however, found that men were more likely to agree with the eradication and control of invasive species than women [31]. We found that for a range of issues regarding weed management in parks, men and women had similar attitudes and experiences. They were equally likely to report having found seeds on their clothing, financially support weed management, agree that visitors unintentionally disperse seeds, and support the cleaning of visitors’ clothing before entering parks.

Although most visitors were concerned about weeds, and many have found seeds on their clothing, over a third reported that they dispose of these seeds in ways that may actually increase the spread of weed in parks. Some visitors mentioned they indiscriminately dispose of seeds on their clothing stating they had previously not given much thought to how they disposed of seeds. These results highlight the importance of education to enhance knowledge of weed hygiene, including the careful disposal of seeds visitors find on their clothing in parks. Information on how to appropriately dispose of seeds could be incorporated into minimum impact codes including in posters, notices or brochures at visitor centres, park entry points and airports as well as applications on mobile devices, and internet based platforms, including social media such as Facebook and Twitter.

In the current study, we provided visitors with five alternative definitions of the term ‘weeds’ with about 75% of visitors correctly selecting the definition of weeds. This could over represent visitor’s knowledge of weeds compared to the types of responses that could be given to an open-ended question on this topic. Future research in the same Park as was surveyed here, as well as in other parks could follow on from the current study by assessing more specific aspects of visitor’s knowledge of weeds and their preferences for different types of management options. It would also be useful to assess visitor’s knowledge of the potential for other types of seed dispersal in parks such as seeds on cars and in the dung of animals such as horses.

## Conclusions

It is widely accepted that the management of weeds in areas of high conservation value such as national parks will not be effective without the support of park visitors. Based on this study, visitors to parks appear likely to support common weed control and eradication programmes as observed in similar studies [27,31,48]. Visitors also appear willing to support spending money on these activities. Other studies [29,30] have shown park visitors are willing themselves to financially support the control of invasive species through park entry fees, and/or indirectly via taxes that are then used to fund park and weed management. Although not assessed here, some visitors may also be willing to directly participate in weed reduction strategies such as volunteering for trail maintenance and restoration events [49]. Visitors could also be encouraged to take personal initiatives that are likely to reduce the spread of weeds in parks. This includes changing what they wear to parks, where they go and what they do if they find seeds on their clothing. For example, changing what one wears, such as covering up socks can reduce the chance of dispersing weed seeds in parks [18,22]. Avoiding walking off track reduces damage to vegetation that favours weeds over native plants. Avoiding walking through weedy verges in car parks prior to starting walks also reduces the chance of weed seeds attaching to clothing [22]. Finally, disposing of any seeds found on clothing in bins will also help limit the spread of weeds, including in parks.

## Supporting Information

S1 Dataset Minimal data set underlying the findings in the study. (ZIP)
